# “Don’t forget the migrants”: exploring preparedness and response strategies to combat the potential spread of MERS-CoV virus through migrant workers in Sri Lanka

**DOI:** 10.12688/f1000research.2-163.v1

**Published:** 2013-07-29

**Authors:** Kolitha Wickramage, Sharika Peiris, Suneth B Agampodi

**Affiliations:** 1 Health Department, International Organization for Migration (IOM), Colombo, Sri Lanka; 2 Department of Community Medicine, Faculty of Medicine and Allied Sciences, Rajarata University of Sri Lanka, Saliyapura, Sri Lanka; 3 Tropical Disease Research Unit, Faculty of Medicine and Allied Sciences, Rajarata University of Sri Lanka, Saliyapura, Sri Lanka

## Abstract

From September 2012 to July 2013, 81 laboratory-confirmed cases of infection with Middle East respiratory syndrome coronavirus (MERS-CoV), including 45 deaths (a case fatality ratio of 55%) have been reported from eight countries. Human-to-human transmission is now confirmed showing potential for another pandemic of zoonotic disease, with an extremely high mortality rate. Effective surveillance strategies are required in countries with a high influx of migrants from the Middle East to mitigate the probable importation of MERS-CoV. We discuss here the risk of MERS-CoV in major labor sending countries and list the probable strategies for control and prevention of MERS-CoV using Sri Lanka as an example. It is conservatively estimated that 10% of Sri Lanka’s population work as international labor migrants (1.8 to 2 million workers), with 93% residing in the Middle East. An average of 720 workers depart each day, with the majority of these workers (71%) departing to the Kingdom of Saudi Arabia (the country with 81.5% of total MERS-CoV cases). We also describe other inbound migration categories such as tourists and resident visa holders relevant to the context of preparedness and planning. The importance of partnerships between public health authorities at national and regional levels with labor migration networks to establish institutional and/or policy mechanisms are highlighted for ensuring effective preparedness and response planning. Strategies that can be taken by public health authorities working in both labor sending and labor receiving counties are also described.  The strategies described here may be useful for other labor sending country contexts in Asia with a high frequency and volume of migrant workers to and from the Gulf region.

## Introduction

The global health community is experiencing one of the deadliest coronavirus outbreaks that has been reported in recent times. The first case of Middle East respiratory syndrome coronavirus (MERS-CoV) infection was reported in September 2012 from the Kingdom of Saudi Arabia (KSA) ^1^. Since then, 81 laboratory-confirmed cases of infection with 45 deaths were reported by eight countries, of which 66 (81.5%) were from the KSA ^2^ ( Table 1). Even though France, Germany, Italy, Tunisia and the United Kingdom have also reported laboratory-confirmed cases, these patients had been either transferred to these countries from hospitals in the Middle East for specialist care or had returned from the Middle East and subsequently became ill. Hitherto, there have been no cases reported in Asia.

**Table 1.** Middle East respiratory syndrome coronavirus – cases and deaths, April 2012 – 11th July 2013 ^2^.

Region and country	Cases	Deaths	Fatality (%)
***Middle East***			
Jordan	2	2	100
Qatar	2	0	0
Saudi Arabia	66	38	57
UAE	1	1	100
***North Africa***			
Tunisia	2	1	50
***Europe***			
UK	3	2	67
France	2	1	50
Italy	3	0	0
***Total***	***81***	***45***	***59***

Coronaviruses have long been known to cause widespread human infections such as the common cold and global pandemics such as severe acute respiratory syndrome (SARS) ^3^. MERS-CoV has not been identified previously among humans ^4^, thus knowledge about the natural history of the disease is still limited. The clinical syndrome of MERS-CoV is primarily a respiratory disease including fever, cough and shortness of breath, resembling SARS. More than half of cases develop life threatening complications, such as respiratory failure ^5, 6^, acute respiratory distress syndrome (ARDS) ^6– 8^, renal failure ^4– 6, 8^, and consumptive coagulopathy ^8^. Studies of clusters of cases suggest that the spread may occur by both large and small aerosols and possibly via the faecal-oral route ^9^. The pathogenesis of MERS-CoV is not fully understood. It appears to cause respiratory problems by attacking and infecting the cells in the nasopharynx; laboratory studies show that the virus has the ability to cause profound apoptosis of human bronchial epithelial cells ^10^. All confirmed cases have had respiratory disease and most have developed pneumonia ^11^. Complications during the course of illness have included severe pneumonia with respiratory failure requiring mechanical ventilations, ARDS with multi-organ failure, renal failure requiring dialysis, consumptive coagulopathy and pericarditis ^11^. Hitherto, 45 out of 81 cases (55%) have died as a result of infection ( Table 1). The rapid transmission and high attack rate in hospital settings have raised concerns about the risk of health care associated transmission of this virus ^12^.

Although the transmission of the disease is still not as rapid as seen during the SARS epidemic in 2003 ^13^, human to human transmission of MRES-CoV has now been established ^5^. Given the high case fatality rate compared to previous coronavirus pandemics, continued risk assessment, surveillance, and preparedness measures by all countries are required to minimize the impact of a probable global pandemic of MERS. The WHO encourages “all Member States to continue their surveillance for severe acute respiratory infections (SARI) and to carefully review any unusual pattern” ^2^.

The annual Hajj pilgrimage, attended by 3 million pilgrims from all over the globe, has been identified as a potential threat for major spread ^14^. A recent study has shown evidence of rapid acquisition of respiratory viruses among pilgrims during their stay during the Hajj in the KSA, most notably rhinovirus ^14, 15^. The authors highlight the potential of spreading these infections in the pilgrims' home countries upon their return. Memish and colleagues also suggest a ‘high degree of clinical vigilance’ required for the possibility of MERS-CoV infection in patients with respiratory infections who have visited the Middle East in the preceding 10 days ^6^. Despite these concerns, the WHO does not recommend changing travel plans for Hajj or Umrah because of MERS-CoV. However, at a recent meeting organized by the WHO in Cairo (June, 2013), public health officials specifically emphasized the importance of preparedness and response at Hajj and contexts of mass gatherings ‘as a priority action’, with Member States of WHO agreeing to develop specific plans for MERS-CoV ^16^. No emphasis at this meeting or in peer-reviewed literature has been made in relation to the large volumes and frequent travel patterns of international labor migrant workers to the Middle Eastern countries, especially from Asia ^17^.

## International labor migrants in the Middle Eastern region

Labor migration from Asia to the Middle East involves the movement of contractual workers from many ‘ *labor sending*' nations such as the Philippines, India, Sri Lanka and Indonesia, to ‘ *labor receiving*' ones, mainly within the Middle Eastern region ^18^. Estimates of total migrant workers by the International Labor Organization for 2010 were 105.5 million, 30 million of which were from within Asia ^19^. It is estimated that there is a net annual outflow of two million migrant workers from the ‘top five’ South Asian labor sending countries of Sri Lanka, India, Bangladesh, Nepal and Pakistan ^20^ ( Table 2). Unregistered ‘irregular’ migrant workers also contribute to this outflow of contractual workers from Asia, although estimates are difficult to assess due to the clandestine nature of their travel. It is important to highlight that remittance from labor migrants contribute significantly to the economic growth of most developing countries in Asia. The Sri Lankan economy is highly dependent on foreign exchange earnings from its migrant workforce, with remittance from workers in Middle Eastern countries alone contributing 58.9% of all total foreign exchange earned in 2011 ^21^.

**Table 2.** Outflow of workers from selected Asian countries to the Gulf Cooperation council countries in 2010 ^26^.

	Labor receiving country						
Labor sending country	Bahrain	Kuwait	Oman	Qatar	KSA	UAE	Total
**Bangladesh**	13,996	29	135,265	13,111	15,039	282,739	*460,179*
**India**	14,323	45,149	73,819	41,710	289,297	138,861	*603,159*
**Nepal**	4,647	15,187	2,442	102,966	71,116	44,464	*240,822*
**Pakistan**	5,940	6,251	37,580	10,171	138,495	222,097	*420,534*
**Sri Lanka**	7,057	48,105	6,370	53,632	70,896	42,198	*228,258*
**Philippines**	15,434	53,010	10,955	87,813	293,049	201,214	*661,475*
**Indonesia**	15,434	45,149	73,819	41,710	289,297	138,861	*603,159*
***Total***	*75,720*	*212,880*	*340,250*	*351,113*	*1,167,189*	*1,070,434*	

## Preparedness measures and screening strategies relevant to Sri Lanka

Although the WHO has not yet issued a travel health warning for any country, nor recommended conducting on-arrival screenings at ports of entry, the infectious nature of MERS-CoV means that there is a risk of contracting the disease through infected individuals who have visited the Middle East in the preceding 10 to 14 days. Health authorities in some countries in the region have already begun making advanced arrangements for the diagnostic test kits developed by the CDC for MERS-CoV to be made available to National Reference Laboratories ^16^. Ensuring guidance for health care professionals regarding case definition, diagnosis and management for MERS-CoV infection, and establishing an active surveillance system for ‘influenza-like’ illnesses in hospitals are essential steps for surveillance. Elaborating pandemic preparedness and response measures are not the focus of this current paper since these have already been well described and indeed established in Sri Lanka through previous efforts against SARS and H1N1 ^22^. Rather, this article will focus on understanding the importance of the large volumes of migration categories and their dynamics, which may yield more specific and targeted public health and screening interventions for MERS-CoV.

## Inbound migration categories to Sri Lanka from the Middle East region

**Figure 1.**
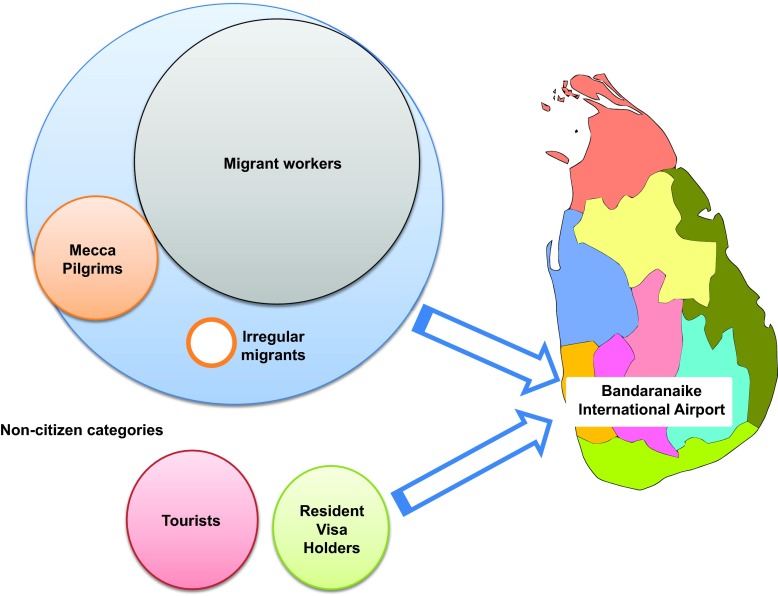
Categories of inbound travelers from the Middle East. This figure shows the different categories of inbound travelers arriving at the Bandaranaike International Airport, Sri Lanka.

Inbound migration refers to the flow of persons traveling into a country ^23^. We identify five major inbound migrant flows from the Middle East to Sri Lanka with the potential of introducing MERS-CoV ( Figure 1).

KSA, Qatar, Kuwait, UAE and Jordan are the major destination (labor receiving) countries, encompassing 85% of Sri Lanka’s total international labor migrant force (1.8 to 2 million workers in 2011) ^21^. Each day, around 720 migrant workers leave Sri Lanka to the Middle East as labor migrants through Bandaranaike International airport ^24^. Over 93% of the 262,960 labor migrants were employed in Middle Eastern countries in the year 2011 ( Table 2). Female participation in foreign employment is 48.3% of the total departures during the same year, and 85% of them worked as domestic housemaid ^25^. The recent evidence of virus spreading within family clusters may be a significant factor in determining household transmission ^6^.

Data on patterns of returning migrant workers are not available since there is no registry of returning workers. However, inflow is expected to be greater than outflow considering both the cyclical nature of labor migration (where a worker usually returns to the country for a short period before departing again - a cycle which can last 10 years or more), and the large stock total of formally registered workers from Sri Lanka.

Every year, Muslims from all over the world converge in KSA to take part in the annual Hajj (pilgrimage). KSA hosted 2.5 million pilgrims in 2009 amidst the H1N1 pandemic ^27^. In 2013, the Hajj is expected to fall between the 13–18 October. A quota system operates to limit the number of people from each country visiting Mecca each year based on the number of Muslims in each country. The Sri Lankan quota for 2013 is currently set at 2,800 ^28^.

### Tourist arrivals and resident visa holders

A residence visa is a permit for non-Sri Lankan citizens to obtain residence facilities for purposes of long stays, work and study. The numbers of both residency visa holders and tourists visiting Sri Lanka from the Middle East, disaggregated by country of residence, are shown in Table 3. Both KSA and the UAE remain the primary source countries of migrants within this inbound category.

**Table 3.** Resident visa holders and tourist arrivals from the Middle East in 2010 and 2011.

	2010		2011	
Country	Tourists	Residents	Tourists	Residents
Bahrain	1,459	3	1,819	2
Iran	1,900	75	2,223	139
Israel	3,919	18	6,164	15
Jordan	1,708	41	1,478	52
Kuwait	2,303	25	2,812	15
Lebanon	1,816	11	1,960	21
Oman	1,359	26	2,177	19
KSA	9,301	20	15,081	51
Qatar	1,574	8	2,788	12
UAE	9,825	18	17,664	18
Egypt	849	62	767	77
Turkey	664	41	1,171	86
Others*	863		1,397	93
**Middle East (Total)**	***37,540***	***441***	***57,501***	***600***

*Others: Yemen, Cyprus, Iraq, Palestine and Syria.

If a highly conservative estimate on the number of labor migrants returning from the Middle East is placed at 220,000 persons per year, then based on data from the five major categories of migrant flows presented here, an estimated 280,901 persons will travel from the Middle East to Sri Lanka. This number does not account for the number of returning Sri Lankan tourists and irregular migrants from the Middle Eastern region. Based on the fact that 71% of the current caseload of Sri Lankan migrant workers depart for the KSA, it is expected that the majority of inbound migrants will be traveling from the same country.

## Preparedness and response strategies for labor migrants

It is important to note that the following recommendations are suggested as a way of enhancing, not substituting, existing frameworks on pandemic disaster preparedness and response. There are currently no established guidelines for MERS-CoV established at country level, unlike in other settings ^29^.

**Figure 2.**
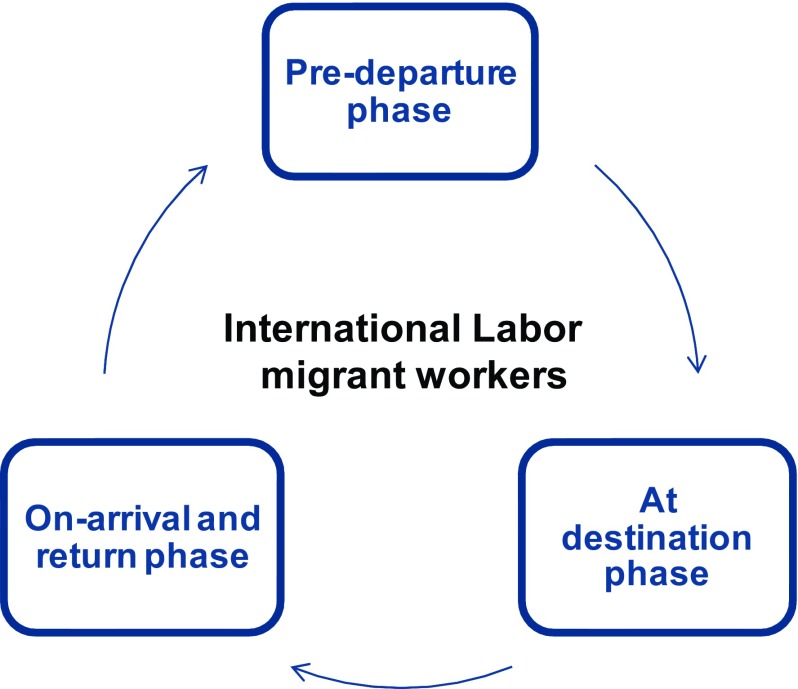
Identifying the ‘intervention space’ within phases of the labor migration cycle. Potential places for interventions (intervention space) in relation to labor migration.

A number of prevention and screening strategies for migrant workers are presented here, classified according to the three phases of migration: ‘pre-departure’ (departing Sri Lanka), ‘at destination’ (time spent in the Gulf States) and ‘upon-arrival’ (arrival back in Sri Lanka). Each stage in the migration cycle offers unique opportunities for public health action/intervention based on enabling mechanisms and capacities harnessed in routine migrant worker pathways ( Figure 2). These may be useful in refining into other country contexts.

**A. Strategies at the 'pre-departure’ phase.** The majority of labor receiving countries require pre-departure health assessment as a pre-requisite for a work visa. Migrant workers to Gulf State countries are expected to undertake a mandatory pre-departure medical examination in Sri Lanka to ensure their ‘fitness to travel’ and fulfillment of health assessment criteria set by the recipient country. Health care workers could provide health information on MERS-CoV to potential migrant workers during the medical examination. The Gulf-Approved Medical Centers Association (GAMCA) has a network of 13 private medical centers in Sri Lanka, which are accredited to conduct health assessments of Sri Lankan migrant workers prior to departure to the GAMCA countries KSA, Kuwait, Bahrain, Qatar, UAE and Oman. As a preparedness measure, medical staff at these health assessment centers can be trained with up-to date information on MERS-CoV and be encouraged to disseminate language specific information-exchange communication (IEC) materials on signs, symptoms and preventative actions for the migrant worker ^30^.

**B. Strategies at the ‘destination’ phase.** Sri Lankan embassies and diplomatic missions at destination countries could disseminate public health service messages in relation to MERS-CoV in Singhalese/Tamil languages via embassy welfare programs, social networks and through ethno-specific radio programs. It is vital that local health authorities and employers provide access for migrant workers to seek primary health care and that they are supported with specialized/referral care within the health system in the Gulf States. The importance of health accessibility, irrespective of visa status, for migrant workers to primary and specialized health care facilities in these destination countries also needs to be emphasized through state-to-state and regional advocacy mechanisms. It is recommended that public health authorities and global bodies such as the WHO and the International Organization for Migration utilize the support of existing inter-regional and trans-national migrant worker networks such as the members of the ‘Colombo process’ and ‘Abu-Dhabi process’ in order to promote effective public health messages and strategies ^31^.

**Figure 3.**
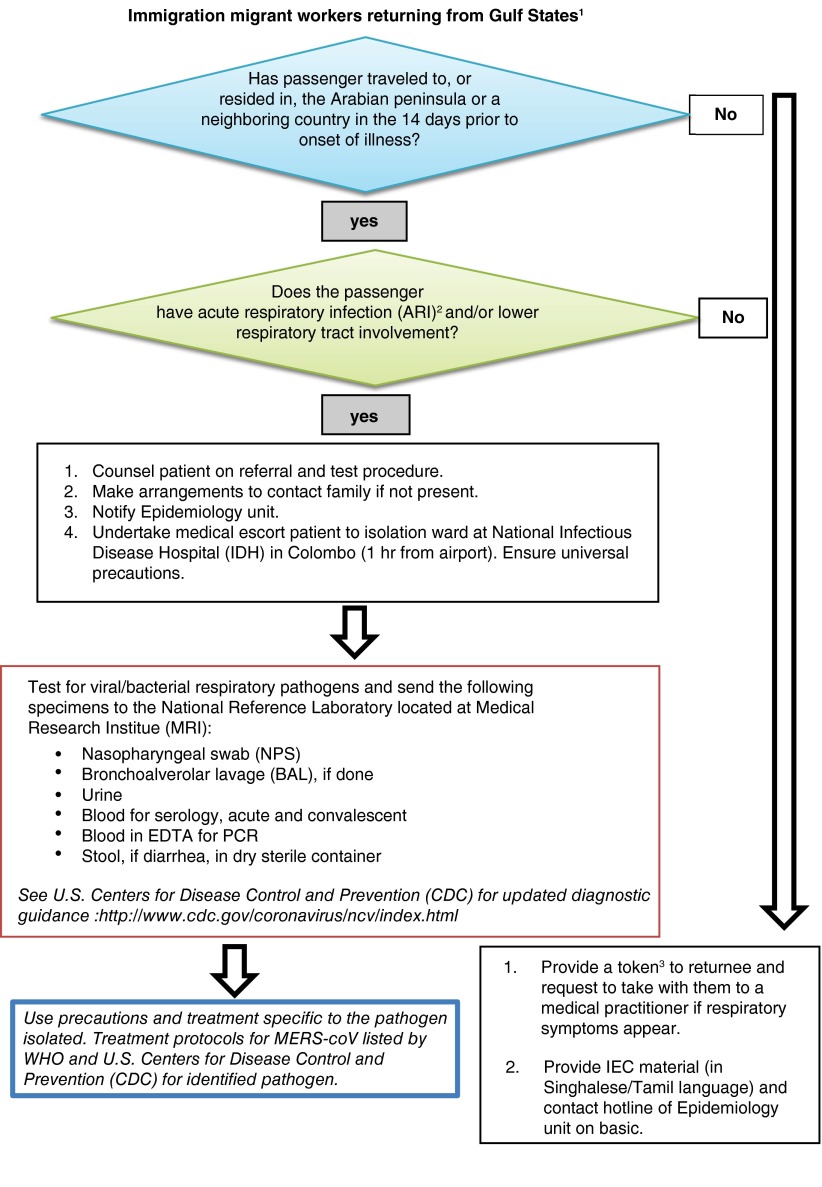
Potential screening algorithm for Middle East respiratory syndrome (MERS-CoV) at Bandaranaike International airport. ^1^Immigration Counter Referral for those migrant workers returning from Arabian peninsula and neighboring Middle East countries (Saudi Arabia, Qatar, Jordan, United Arab Emirates, Bahrain, Iran, Iraq, Israel, Kuwait, Lebanon, Oman, Palestinian Territories, Yemen, Syria). Referral to Airport health unit may also be directed from the SLFBE migrant worker arrival desk. ^2^Acute Respiratory Infection (ARI): Any new onset acute respiratory infection that could potentially be spread by the droplet route (either upper or lower respiratory tract), which presents with symptoms of a new or worsening cough or shortness of breath and often fever (>38°Celsius). ^3^This token will identify the migrant worker as a susceptible person for MERS-CoV.

**C. Strategies at the ‘on-arrival’ phase.** The Sri Lanka Bureau of Foreign Employment (SLFBE) which provides policy direction and regulation of labor migrants has a dedicated 24-hour administrative desk at Sri Lanka’s Bandaranaike International Airport, to manage grievances from returning migrant workers. A worker welfare center to house migrant workers in need of support managed by the SLFBE is also available near the airport. Currently there are no medical personnel attached to the SLFBE services for on-arrival phase. It is recommended that the Ministry of Health make arrangements to establish a coordination mechanism with the SLFBE and with airport health authorities, which currently have no linkage to migrant worker programs. A rotating roster of trained health professionals allocated at the health center at the airport could ensure each returning worker completes the rapid symptom checklist (see assessment algorithm in Figure 3). The algorithm was developed after augmenting the guidance frameworks for MERS-CoV created by the public health authorities in Canada ^29^ and the CDC ^32^. It is important for port health authorities to also build effective partnerships and protocols with immigration control officers at ‘on arrival counters’. This will ensure referral of travelers returning from the Middle East where cases of MERS-CoV have been reported to the health screening desk. Leaflets advising travelers of symptoms of the influenza-like illness could also be distributed at the immigration counter to arriving passengers.

Managing risk communication also forms a vital strategy for any form of public health preparedness and response. Studies have shown that when responding to a novel infectious disease outbreak, policy and planning decisions can limit the ability to control the outbreak and result in unintended consequences including lack of public confidence ^33^. Communication of risk to target populations needs to be carefully planned to avert maladaptive behaviors due to fear and defensive avoidance (the motivated resistance to the message, such as denial or minimization of the threat ^34^). Individuals may defensively avoid a message by being inattentive to the communication (e.g., looking away from the message), or by suppressing any thoughts about the threat over the long term. Mitigating such threats through targeted communication strategies to migrant workers and other categories such as those described above may be useful ^35^. The strategies outlined above do not warrant large scale ‘national level’ awareness campaigns, which may exacerbate anxiety and induce maladaptive rather than positive health seeking behaviors ^36^.

## Conclusion

It has been one year since MERS-CoV was discovered, yet many questions remain unanswered about its pathogenesis, host reservoirs and transmission dynamics. What is clear from global health authorities is that countries need to plan for preparedness and response planning ^29^. We recommend partnerships between public health authorities, at national and regional levels, with the labor migration industry and migrant worker networks in establishing both institutional and policy mechanisms to ensure effective preparedness and response planning in response to a potential MERS-COV threat through labor migrants from South Asia.
