# Supplementary material for: “Don’t forget the migrants”: exploring preparedness and response strategies to combat the potential spread of MERS-CoV virus through migrant workers in Sri Lanka
Source: F1000Res. 2013 Jul 29;2:163. [Version 1] doi: 10.12688/f1000research.2-163.v1 (PMC3886786; doi:10.12688/f1000research.2-163.v1)
Supplement: Peer review history

# Peer review: Referee response for version 1

**Published:** 2013-11-11

**Authors:** Maria van Kerkhove

**Affiliations:** 1 Faculty of Medicine, School of Public Health, Imperial College London, London, UK

I only have one major edit that I would recomend for this article; Table 1 is not necessary and can be removed.

I have read this submission. I believe that I have an appropriate level of expertise to confirm that it is of an acceptable scientific standard.

# Peer review: Referee response for version 1

**Published:** 2013-08-19

**Authors:** Christian Gericke

**Affiliations:** 1 Wesley Research Institute, Brisbane, Austria

Well written paper the spreading potential of MERS-CoV through migration in Sri Lanka.

I have read this submission. I believe that I have an appropriate level of expertise to confirm that it is of an acceptable scientific standard.

# Peer review: Referee response for version 1

**Published:** 2013-08-06

**Authors:** Kayvon Modjarrad

**Affiliations:** 1 National Institute of Allergy and Infectious Diseases, NIH, Bethesda MD, USA

The current manuscript, submitted by Wickramage *et al*., comments on the potentially important role migrant workers may play in the evolving MERS-CoV epidemic. The authors focus most of their discussion on laborers traveling between Sri Lanka and countries bordering the Persian Gulf where most MERS cases have been identified. The authors raise a couple of important concerns: 1) Migrant laborers could serve as a major vehicle for the international spread of the MERS coronavirus; 2) MERS-specific surveillance strategies are needed, particularly for Southeast Asian countries that receive large numbers of returning laborers from the Middle East. 

 These points can be presented, however, with much greater clarity and brevity. The authors occasionally use hyperbole and repetition to make misleading assertions. For example, "the extremely high" mortality rate they cite has been falling as more cases are identified and will likely continue along this trend as milder cases are discovered. Claims of "rapid transmission" and a "high attack rate" are made without any epidemiologic basis or support from literature. Throughout the commentary, the authors also make redundant, disorganized, and extra-contextual statements about MERS clinical features, labor sending/receiving nations, the Hajj pilgrimage, and strategies for controlling a potential MERS epidemic. The only way this manuscript can be salvaged is if it is refashioned as a brief communication of no more than a few hundred words with one figure or table. The manuscript should be narrowly focused on migrant workers between Gulf States and Southeast Asia and the threat they may pose to the spread of the virus. The additional commentary on screening and surveillance adds nothing to the existing WHO report and essentially boils down to education, awareness, and appropriate referral.  The following are specific points of concern:

- Instead of presenting the number of cases and deaths in each country in tabular format, the authors  should provide a link to WHO’s running tally as the authors numbers will be (as they are now) inaccurate and out-of-date. 
-  Remove alarmist statements such as “extremely high mortality rate”, “deadliest coronavirus outbreaks” and platitudes such as “the infectious nature of MERS-CoV means that there is a risk of contracting the disease through infected individuals”
  . 
- I’m confused by the last sentence of the introduction’s first paragraph: “there have been no cases reported in Asia.” 
- The second paragraph of the introduction repeats the clinical features of MERS. This should be corrected. 
- The Hajj pilgrimage is discussed briefly in the introduction, dropped, and then brought up again without any context later in the manuscript. The same is true regarding labor migration patterns and surveillance strategies. There is no real coherent flow to any of these topics.
- Figure 1 and 2  - no information to what is stated in the text. 
- The terms created for the different phases of labor migration cycle make no sense. To the best of my understanding, “pre-departure” means departure and “upon-arrival” means return. These terms are superfluous and are more jargon than they are informative. 
-  Figure 3 is unclear. Is this for anybody returning from a Gulf State or only those returning with symptoms? The first diamond makes this unclear.

I have read this submission. I believe that I have an appropriate level of expertise to confirm that it is of an acceptable scientific standard, however I have significant reservations, as outlined above.
